# Predictability of Dental Distalization with Clear Aligners: A Systematic Review

**DOI:** 10.3390/bioengineering10121390

**Published:** 2023-12-04

**Authors:** Angelo Michele Inchingolo, Alessio Danilo Inchingolo, Vincenzo Carpentiere, Gaetano Del Vecchio, Laura Ferrante, Angela Di Noia, Andrea Palermo, Daniela Di Venere, Gianna Dipalma, Francesco Inchingolo

**Affiliations:** 1 Department of Interdisciplinary Medicine, University of Bari Aldo Moro, 70124 Bari, Italy; angeloinchingolo@gmail.com (A.M.I.); ad.inchingolo@libero.it (A.D.I.); vincenzo.carpentiere@gmail.com (V.C.); dr.gdelvecchio@gmail.com (G.D.V.); lauraferrante79@virgilio.it (L.F.); angeladinoia@libero.it (A.D.N.); daniela.divenere@uniba.it (D.D.V.); francesco.inchingolo@uniba.it (F.I.); 2 College of Medicine and Dentistry, Birmingham B4 6BN, UK; andrea.palermo2004@libero.it

**Keywords:** clear aligner therapy (CAT), tooth movement, malocclusion, orthodontics, molar distalization, alignment

## Abstract

The current review aims to evaluate the scientific evidence relating to the effectiveness of treatment with clear aligners (CAs) in controlling distalization orthodontic tooth movement. “Orthodontics, aligners” and “distalization” were the search terms used on the Scopus, Web of Science and Pubmed databases with the Boolean operator “AND”. The results of the last ten years of research were 146 studies; of these, 19 publications were included for this review. The distalization movement is possible with invisible masks alone, but the risk of losing anchorage in the anterior sectors is very probable. The stability of the results and the reduction of unwanted effects can be guaranteed by the use of skeletal anchoring devices and interproximal enamel reduction (IPR), with which compensations are obtained to reduce the initial overjet. Temporary anchorage devices (TADs) can be used to manage posterior anchorage after distalization of maxillary molars with aligners. This hybrid approach has demonstrated the greatest orthodontic success. TADs are useful aids to provide direct and indirect skeletal anchorage. The opposite effect must be considered when planning dental distalization, especially of the molars, in patients with large overjet, and corrective measures or the use of auxiliaries may be necessary to prevent midcourse corrections. This systematic review provides a critical evidence-based assessment of the predictability of dental distalization with CAs, an ever-evolving orthodontic technique.

## 1. Introduction

The correction of orthodontic defects and dental malocclusions is a crucial objective to improve masticatory function and smile aesthetics [1,2]. Over the years, changes in the field of orthodontics have been notable and have followed the transition from traditional metal braces to more modern and aesthetically acceptable solutions [3,4,5]. They have opened new possibilities [6,7,8,9]. With the introduction of digital methods, orthodontics has seen an amazing advancement in technology. Image processing procedures have been expedited even more by the incorporation of deep learning into software. When doctors employ artificial intelligence, their diagnosis, treatment planning, growth and development evaluation, treatment progress and result assessment, maintenance phase, remote monitoring, and long-term follow-up all improve. Improvement refers to the potential for a more efficacious and comprehensive integration of the clinically chosen and gathered data [10].

Data collection from a range of sources, like dental radiography, digital models, clinical data, remote monitoring, and devices like cone beam computed tomography (CBCT), has been made feasible by the advancement of digital technology in dentistry.

The advancement of technology has made it feasible to improve orthodontic diagnosis, treatment planning, and long-term follow-up by utilizing tools like data science, machine learning, and cloud-based systems. We stress how crucial it is that medical professionals learn how to use AI-powered orthodontic imaging technologies.

Although dental and craniofacial connection analysis can be improved with the use of artificial intelligence (AI) technologies, it is imperative that physicians combine AI with thorough analysis rather than depending exclusively on automated [10,11]. 

When clear thermoplastic sheets were first employed in orthodontics in the 1980s, they were primarily utilized as retainers, but it was quickly realized that they could also realign teeth. These days, they are referred to as “clear aligners”. Depending on the degree of tooth misalignment, each patient will receive a unique set of braces that are completely undetectable and removable [12]. Initially, they were used for their ability to discreetly and effectively correct some dental malocclusions, for the correction of small dental crowding or space closures, and subsequently for the correction of movements that are more difficult to obtain, such as dental distalization [13,14,15]. However, one of the main factors that contributes to the success of any orthodontic treatment is the predictability of the results [16,17].

**Figure 1:**
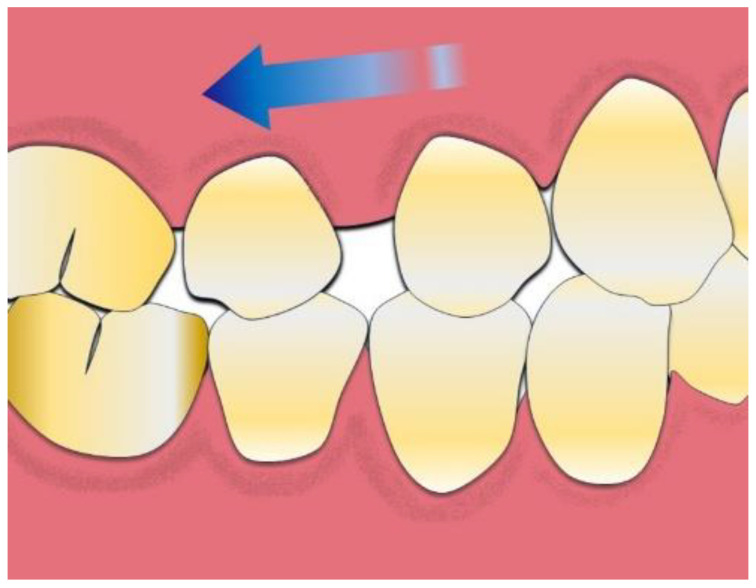
Dental distalization movement is illustrated. The arrow indicates the direction of tooth distalization.

Dental distalization is an objective of the orthodontic treatment plan that aims to move the posterior teeth in a distal direction in the upper or lower dental arch to expand the arch and achieve distalization of the frontal group [18,19,20]. This movement is necessary in the treatment plan, in the second dental or skeletal classes with increased overjet, to avoid carrying out extractions in the upper arch, or in the third classes, to solve orthodontic problems, such as crowding and malposition of the molars (Figure 1) [21,22,23]. 

Traditionally, dental distalization was achieved with the application of extraoral devices, such as the EOF (extra-oral force), which requires the patient’s collaboration, or using intraoral devices such as the distal jet, the pendulum, etc. [24,25], which do not require the patient’s collaboration and are fixed appliances made up of wires and brackets [18,19,26]. However, currently, transparent aligners, also known as transparent alignment devices, represent a valid and increasingly used alternative for this purpose [27,28,29].

**Figure 2:**
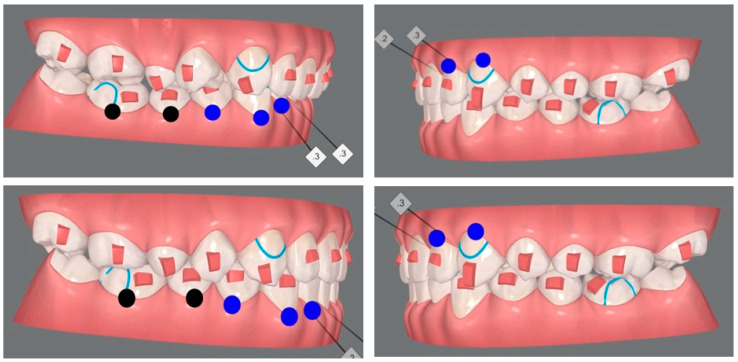
ClinCheck simulation of orthodontic distalization is illustrated.

CAs are made of clear plastic material and are made using a digital impression of the patient’s jaws, by ClinCheck software, a machine that converts the data taken from the impression into a series of individual aligners, which perfectly fit the patient’s teeth (Figure 2) [27,30]. 

Technological innovation is at the center of attention regarding therapy with transparent aligners [31]:

1. The material used for the production of the masks is highly innovative; polyurethane is generally used, and even more recently, a multilayer aromatic thermoplastic polyurethane/copolyester, which despite being very thin, guarantees greater resistance and flexibility, and is also hypoallergenic, inert and biologically stable [32].
2. Increasing efforts are being made in digitalization using the latest technology to diagnose each case precisely and professionally. During the first visit, a complete 3D scan is performed to see the general state of the oral cavity. Furthermore, it is possible to carry out a video simulation so you can see what the final result will be like before starting orthodontic treatment [33,34].
3. Another innovation consists in the use of a 3D printer to produce the aligners [35].

Ultimately, we can highlight that the advent of digital systems, from CBCT to intraoral scan and the software connected to these devices, has speeded up and made the process of diagnosing an orthodontic case more precise. The integration of deep learning within the software has further accelerated the processes of image processing [36,37].

These devices apply gradual pressure on the teeth, pushing them into the desired position over time [38,39,40]. However, their effectiveness and, more importantly, their predictability in achieving orthodontic goals remain the subject of research and discussion [41]. The predictability of orthodontic treatment is a key aspect for patients and orthodontists [42,43,44]. CAT promises predictable results, but its ability to accurately achieve dental distalization goals has been the subject of ongoing scientific investigation [45,46,47].

Understanding the predictability of dental distalization with CAT is critical for several reasons [41,48]. First, it can help orthodontists make informed decisions about orthodontic treatment and communicate effectively with patients based on their expectations and the results that can be achieved [49,50,51]. Secondly, greater treatment predictability can reduce the risk of delays and complications, thus improving the overall efficiency of the orthodontic process [52,53,54]. Finally, a better understanding of predictability may influence patients’ choice of treatment, as a more predictable treatment may be considered more attractive [9,49,55]. Keep in mind that using aligners needs a lot of patient cooperation. As a result, orthodontic outcomes depend not only on the clinician’s operational and planning abilities but also on the patient’s cooperation [56]. There is yet another aspect to consider. Although orthodontic aligners are comfortable and aesthetic, due to the possibility of bisphenol A (BPA) leakage, resulting in cytotoxicity, adverse effects and estrogenic effects, the biomaterials used in these devices could be hazardous for biosafety and biocompatibility [57]. It appears that the safety of these devices may be called into question due to these levels of BPA, even at low doses, as well as due to the numerous adverse events associated with clear aligners or clear retainers, such as soft tissue issues, such as burning, tingling, swelling of the lips, blisters, ulceration, dry mouth, periodontal problems and, last but not least, difficulty breathing and problems related to oral dysfunction, linguistic disorders and dental damage, which are all aspects that should be taken into consideration [58,59]. Further investigations regarding the biocompatibility of these devices are therefore necessary, particularly when remembering that even these CAs, although so widespread, have limitations. In particular, the aligners are not able to intervene on the transverse plane, and are therefore not able to exert those orthopedic effects necessary, for example, for palatal expansion through the opening of the palatine suture, which is instead possible through traditional equipment, whose effects are unequivocally described in the literature. It must absolutely not be forgotten that transparent masks cannot completely replace traditional methods; rather, they must integrate and work synergistically with them [60,61,62].

## 2. Materials and Methods

### 2.1. Protocol and Registration

This systematic review was conducted by the Preferred Reporting Items for Systematic Reviews and Meta-analysis (PRISMA) standards and submitted to PROSPERO with number ID: CRD-487956. 

### 2.2. Search Processing

Orthodontics, aligners and distalization were the search terms utilized on the databases (Scopus, Web of Science, and Pubmed) to select the papers under evaluation, with the Boolean operator “AND”.

The search was restricted to just items released in the English language during the previous ten years (2013–2023) (Table 1). 

### 2.3. Eligibility Criteria

The reviewers (V.C. and L.F.), who worked in pairs, chose works that satisfied the following criteria for inclusion: (1) human studies; (2) clinical studies or case reports; (3) in vivo studies.

Exclusion criteria were systematic reviews, meta-analyses, animal studies, no English language and in vitro studies.

### 2.4. Data Processing

The screening process allowed for the removal of any publications that did not fit the topics examined. It was carried out by reading the article titles and abstracts selected in the previous identification stage.

After being found to meet the predefined inclusion criteria, the full text of the publications was reviewed.

Disagreements among reviewers on the selection of the article were discussed and resolved.

### 2.5. PICOS Criteria

The PICOS (Population, Intervention, Comparison, Outcome, Study Design) criteria, which are used in this assessment, are represented in Table 2 as population, intervention, comparison, outcomes, and study design.

#### Quality Assessment

The quality of the included papers was assessed by two reviewers, RF and EI, using the reputable Cochrane risk-of-bias assessment for randomized trials (RoB 2). The following six areas of possible bias are evaluated by this tool: random sequence generation, allocation concealment, participant and staff blinding, outcome assessment blinding, inadequate outcome data, and selective reporting. A third reviewer (FI) was consulted in the event of a disagreement until an agreement was reached.

## 3. Results

Keyword searches of the Web of Science (29), Scopus (21) and Pubmed (96) databases yielded a total of 146 articles. The subsequent elimination of duplicates (32) resulted in the inclusion of 114 articles. Of these 114 studies, 90 were excluded because they were off topic.

**Figure 3:**
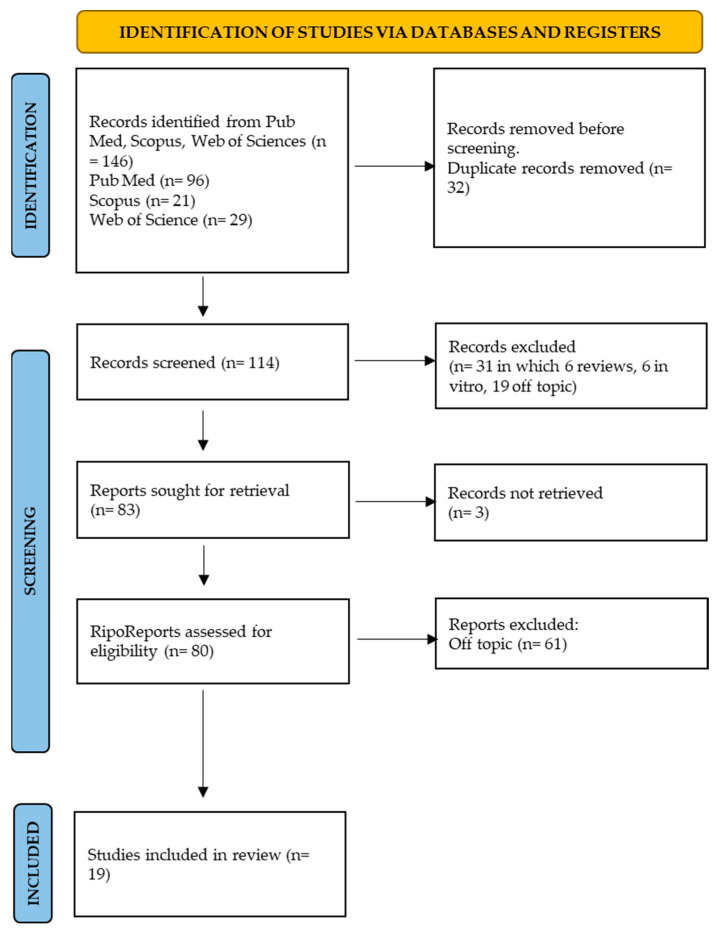
Prisma Flow chart.

The screening phase ended with the selection of 19 publications for this work (Figure 3). The results of each study are reported in Table 3.

### Quality Assessment and Risk of Bias

**Figure 4:**
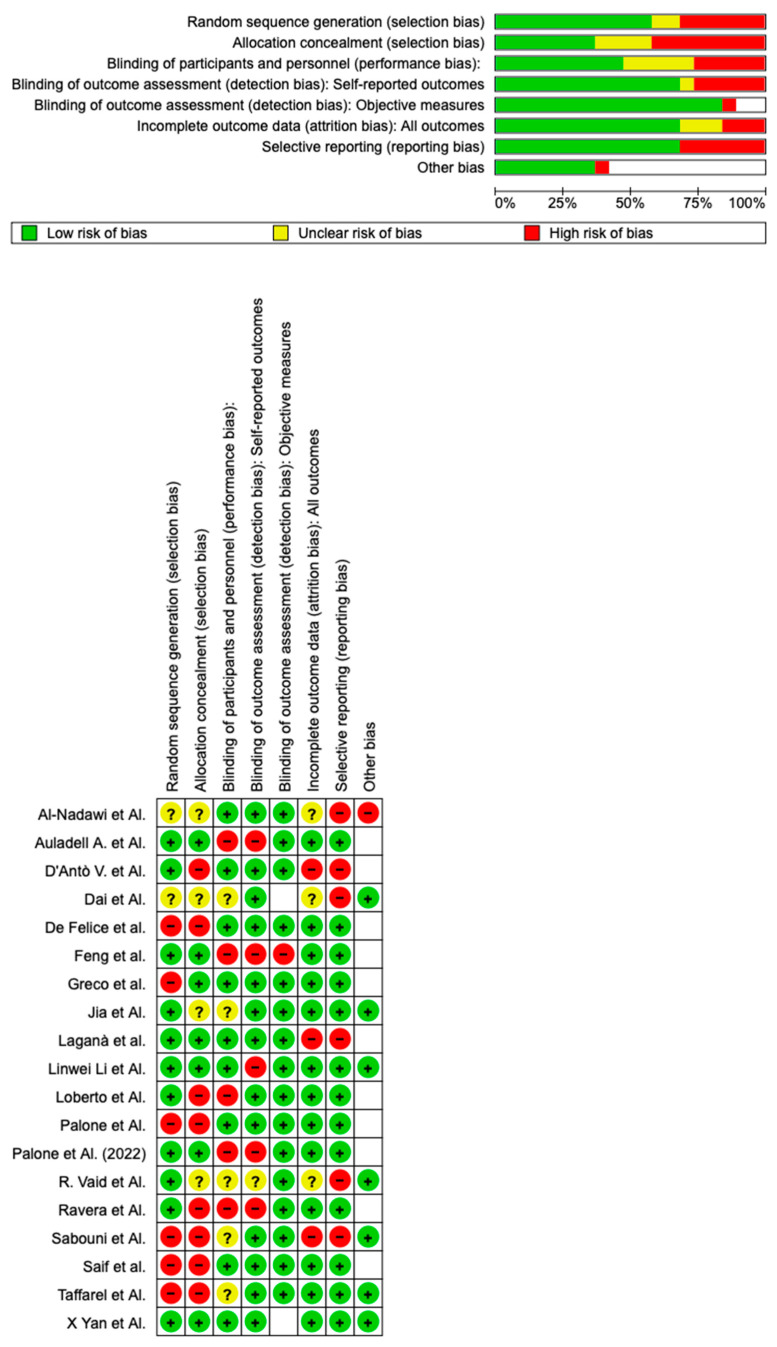
Risk of bias is illustrated; red indicates high risk, and green indicates low risk of bias. Judgment: + low concerns; ? no informations; − very high concerns [1,15,24,25,26,27,28,29,30,31,32,33,34,35,36,38,39,40].

The risk of bias in the included studies is reported in Figure 4. Regarding the randomization process, 50% of studies present a high risk of bias and allocation concealment. All other studies ensure a low risk of bias. A total of 75% of studies exclude a performance; half of the studies confirm an increased risk of detection bias (self-reported outcome), and 75% of the included studies present a low detection bias (objective measures) (Figure 4). A total of 75% of studies ensure a low risk regarding attrition and reporting bias.

## 4. Discussion

**Figure 5:**
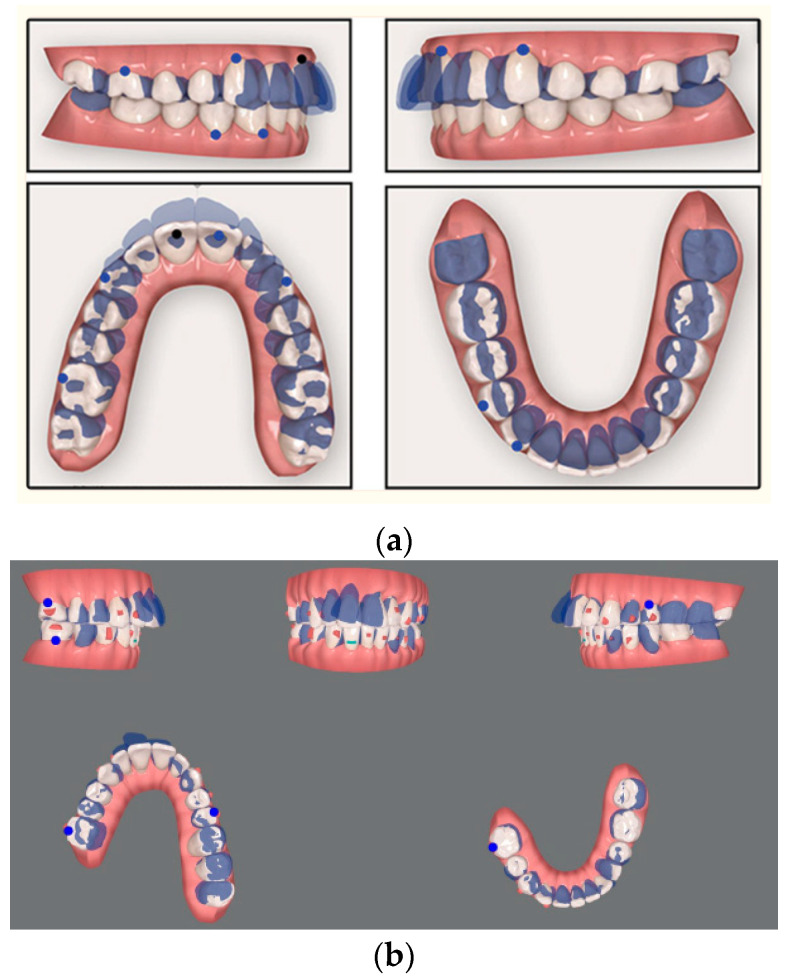
Examples of superimposition of pre- (**a**) and post-treatment (**b**) digital models of orthodontic clinical cases are presented, via digit.

Over the past 20 years, technological advances have transformed orthodontics by creating systems of CAs as alternatives to fixed braces, capitalizing on patient demands for aesthetics and customization. CAs are clear, removable orthodontic devices that are used to gradually move teeth into the desired position. They are an aesthetically pleasing alternative to traditional fixed orthodontic appliances, as they are barely visible [27,74]. The benefits of CAs include improved aesthetics, patient comfort, ease of oral hygiene, reduced risk of periodontal problems and enamel decalcification, reduced occlusal abrasion, less frequent appointments, and reduced chair time. The success of the treatment always depends on the patient’s motivation in wearing the aligners for most of the time in each 24 h period and on the operator’s ability to correctly plan a treatment plan most suitable for the patient. Distalization of the maxillary molars is a common treatment, especially in Class II molar malocclusion, which involves distalization of the upper teeth to correct the molar relationship. This treatment is recommended for patients with maxillary dentoalveolar protrusion or minor skeletal discrepancies. Traditional distalization devices include removable extraoral anchoring devices and fixed intraoral anchoring systems [40,79,80]. Over time, fixed intraoral distalization devices with dental anchoring have been developed [72,81]. However, some negative effects may occur during therapy, such as loss of dental anchorage, widening of the maxillary incisors, mesialization of the premolars and increase in the lower anterior facial height. CAT is often used to treat Class II malocclusions when distalization and derotation of the upper first and second molars are viable options [72,82]. The therapists’ intended treatment outcome might not be reached despite the scant evidence supporting these movements’ predictability. Determining the precision of distalization and derotation with CAs is the aim of this work. The pre-treatment, post-treatment, and virtual plan (ideal post-treatment) measurements of 16 patients (4 M, 12 F; mean age 25.7, 8.8 years) were superimposed using digital models using the 3D quality control program Geomagic Control X. The amount of tooth movement suggested and achieved was calculated using linear and angular measuring instruments [41]. The buccal cusps had been dislodged proximally (Figure 5) [41].

The findings showed that when the anticipated movement is around 2.6 mm, aligners are useful for molar distalization. However, a significant difference was found between the predicted and actual movement of the molars. Furthermore, a correlation was observed between the distal molar movement and loss of anterior anchorage [83]. Therefore, the reverse effect must be considered when planning molar distalization, especially in patients with large overjet, and that corrective measures or the use of auxiliaries may be necessary to prevent midcourse corrections [41,84]. The accuracy of distalization and derotation of upper first and second molars with transparent aligners was evaluated by D’Antò et al. in 2022. The aligners demonstrated an overall accuracy of 69% for distalization of the buccal cusps of the first molars and 75% for the second molar. The study by Li et al. in 2022 evaluated the effectiveness of distalization of upper molars with or without retraction of anterior teeth using CAs, and the results showed that the effectiveness of distalization of molars was significantly influenced by the retraction of anterior teeth, with greater effectiveness in patients without retraction [69]. Furthermore, expansion of the dental arch was observed in patients without retraction. Ravera et al. examined in 2016 the possibility of distalizing upper first molars using CAs in combination with Class II composite and elastic brackets. The results indicated that distalization of the first molars can be achieved without significant inclination or vertical movement [70]. According to a study carried out by Sabouni et al., in 2023, the combined use of aligners with appropriate position and attachment geometry is an effective means of solving more complex orthodontic problems such as Class II malocclusions in a comparable time frame, if not inferior to conventional fixed orthodontics but with excellent aesthetics, oral hygiene and quality of life [66,85]. CAT, in a study model by Jia et al., with 3D anchor attachments, could be effective in controlling the rotation and tipping of the anchor units caused by the 3D anchor attachment, which confirmed that the CA could improve root movement [67,86].

### 4.1. Time of Use

In orthodontic treatments with CAT by Al-Nadawi et al. 2021, 7-day and 14-day usage protocols were taken into consideration and analyzed separately [68]. The 14-day wearing protocol showed statistically greater accuracy for some posterior tooth movements: maxillary intrusion, buccal crown torque, as well as mandibular intrusion and extrusion [68,87]. None of them went over the level that is clinically important (>0.5 mm or >2°). The 7-day procedure is an appropriate treatment regimen, as evidenced by the fact that it achieved clinically equivalent accuracy to the 14-day program in half the treatment period. The 14-day regimen should be taken into consideration, nevertheless, if difficult posterior tooth motions or angular movements (such as torque, tip, and rotation) are necessary [68]. The findings are somewhat consistent with those of Simon et al., who discovered that molar distalization was foreseeable as a linear motion [68,88].

### 4.2. TADs

A study by Loberto et al. in 2023 examined the results of CAT in the distalization of upper molars [72]. Significant distalizations of the maxillary first permanent molars (2.5 mm) and significant mesial displacements of the maxillary canines (1.33 mm) were found, with therefore promising results [89,90]. However, a slight loss of anchorage was observed during treatment [72]. Temporary anchoring devices (TADs), which use bone rather than teeth as the anchoring unit with beneficial results in the distalization of maxillary molars, have recently been adopted in orthodontic therapy to avoid unwanted secondary tooth movements [91,92,93]. However, even when using TADs, it is important to take into account some issues such as screw fracture, pain and bulk, which lead to a lack of cooperation. Orthodontic treatment with dental distalization movement with removable transparent aligners, CAT, seems to have excellent results in association with TAD [94]. Palone et al., in 2022, explain how maxillary skeletal contraction is a common problem in adults and can cause aesthetic and functional problems. The rapid jaw expander is an effective treatment to correct this condition in young patients but becomes less effective with age [73,95]. The use of a traditional expander in adults may primarily involve tooth expansion and may have periodontal side effects with unstable results and a high likelihood of recurrence [73,96]. Skeletal anchorage using TADs is an effective method for correcting Class II malocclusions in adults without the need for extraoral devices and with little risk of loss of anterior anchorage. The use of transparent aligners is ideal in the finishing phase of an orthodontic treatment, despite some limitations in tooth movement [73,97]. The hybrid approach, with the use of a skeletally anchored maxillary expander and distalizing appliance, followed by transparent aligners to complete the orthodontic treatment of an adult patient, has also proven to be particularly effective [73]. The use of TADs may be useful to manage posterior anchorage after distalization of maxillary molars with aligners, also according to Greco et al. (2022) [78,98]. This hybrid approach, called “G-block,” has demonstrated success in treating a Class II malocclusion. TADs were placed between the maxillary first and second molars to provide direct and indirect anchorage [99]. This approach resulted in a solid Class I occlusion, improving esthetics and reducing the need for elastics, thus simplifying the treatment of posterior teeth and improving orthodontic control [78]. Evaluating the possibility of distalizing the lower molars using transparent aligners and mini implants as anchorage was the aim of a study by Auladell et al. in 2022 who concluded that, for distalizations greater than 3 mm, there is no predictable protocol. The use of mini implants in combination with aligners can improve the predictability of lower molar distalization [1].

### 4.3. IPR

To address dental crowding problems, in combination with CAT, a simple procedure called interproximal enamel reduction (IPR) is very often used, which is a procedure that involves the controlled removal of the enamel between the teeth to create space and facilitate the movement of the teeth during orthodontic treatment [74,100]. IPR is indicated in some specific situations, such as when you need to create space between crowded teeth. However, there are also contraindications, for example in cases of dental hypersensitivity, active periodontal disease and other specific conditions [74,100]. De Felice et al., in 2020, presented a study aimed at verifying the accuracy of the amount of enamel removed during IPR which can vary considerably compared to what was initially planned via digital set up. In some cases, the actual amount of IPR performed may be less or, in rare cases, more than expected in the treatment plan, and in many cases the difference may not be clinically relevant. The variability depends on several factors, including the IPR technique used, the hardness of the enamel and the experience of the operator [74,101]. Therefore, the accuracy of IPR in CAT is an aspect to consider in improving orthodontic outcomes with this technique [74]. Through the IPR (interproximal enamel reduction) performed by the orthodontist during treatment with transparent aligners, compensations are obtained to reduce the initial overjet. The results demonstrated a correspondence between the amount of enamel planned and that removed in vivo, confirming the reliability of the ClinCheck software [74,77,102]. This suggests that digital planning can be used with confidence to guide IPR during aligner treatment, providing accurate results [77]. In Palone’s 2023 study, it is recalled that the average effectiveness of CAT is 41%. To achieve a more efficient CAT, you need to add approximately 20% overcorrection to the initial planning phase when planning challenging movements such as tilt and rotation. The amount of overcorrection, however, to be added to the initial planning depends on the amount of movement prescribed and the type of tooth involved [71,103]. This information is valuable for improving the effectiveness and efficiency of CAT, reducing the need for additional refinement steps and making the treatment more economical and rapid [71].

### 4.4. Extraction

**Figure 6:**
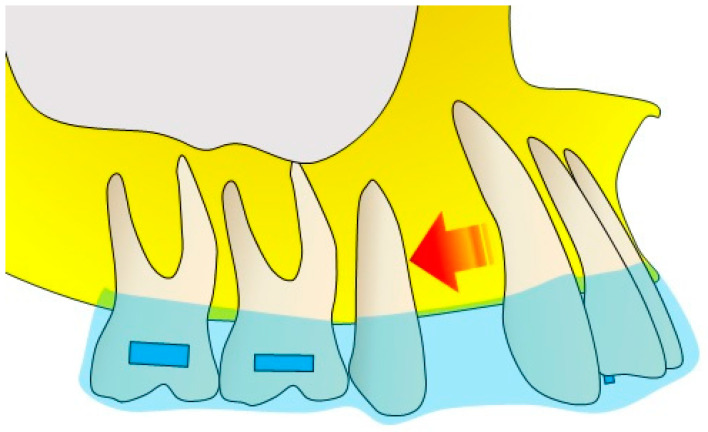
Distalization of the canine in an orthodontic treatment with CAT, with extraction of the first upper premolar, is presented. The arrow indicates the direction of tooth distalization.

To have a good orthodontic result, all the teeth, at the end of the treatment, must be correctly aligned for a stable and functional occlusion and for an optimal aesthetic appearance [75,104]. A study by Feng et al., in 2022, addressed the problem of unwanted movement of teeth adjacent to the extraction site during orthodontic treatment [75]. From a biomechanical point of view, CAs produce an orthodontic force that moves the teeth thanks to the reversible deformation of the aligner and a certain level of elasticity [105]. The use of transparent aligners to close an extraction site can cause a pronounced inclination of the adjacent teeth, such as the canines, in the case of extraction of the first premolar, and the posterior teeth, towards the extraction space [75,106]. It has been observed that a certain level of computer planning on the transparent masks can prevent unwanted rollovers towards the extraction space during the closure of the space [75,107]. The extent of the anti-tipping design to be planned is related to the amount of distalization of the canine that you want to obtain, in the case of extraction of the first premolar (Figure 6), to the amount of movement of the posterior teeth, to the reduced length of the dental arch and to the initial mesiodistal inclination [38]. These results and formulas could be used as a useful guide to improve planning [108].

According to research performed by Dai et al. [63], people who underwent four first premolar extraction procedures with Invisalign could not entirely accomplish the desired coronal motions of their first molars, canines, and central incisors [63]. Greater mesial inclination, buccal inclination, mesio-lingual rotation, mesial displacement and intrusion, and less constriction than anticipated were accomplished by the first molars [63,109]. The central incisors and canines had less retraction and intrusion than anticipated, as well as higher distal tipping and lingual inclination [63,109]. The maxilla and mandible showed different variations in these discrepancies between the achieved and projected tooth crown movement [63]. Vaid et al. demonstrated that CAs are biomechanically inadequate to achieve complex orthodontic movements based on the use of aligners alone, and clinical success depends on the clinical experience of the orthodontist [64]. Taffarel et al. also superimposed the clin check and final clinical outcome of orthodontic cases in adults [65]. They concluded their study by writing that distalization was not achieved [65,110]. CAT can partially achieve incisor proclination (69.8%) and intrusion (53.3%), but excessive labial movement of the incisors may occur as a side effect that should be prevented, as demonstrated in a randomized study by Yan et al. [2]. Unfortunately, the research has limitations relating to the limited literature regarding distalization with CAT; therefore, further studies are necessary to evaluate this recently used method [84].

## 5. Conclusions

CAT is an effective procedure capable of aligning and levelling the dental arches even in non-growing subjects. It is effective in controlling the body movement of the upper molars when a distalization of 2.6 mm has been prescribed. The reverse effect should be considered when planning molar distalization, especially in patients with large overjet, and that corrective measures or the use of auxiliaries may be necessary to prevent midcourse corrections. The aligners have demonstrated an overall accuracy of approximately 70% for distalization of the buccal cusps of the first and second molars. The 14-day CAT use protocol showed statistically greater accuracy for some posterior tooth movements than the only 7-day use protocol. The use of CAT requires not only aligners but also the use of auxiliary devices such as brackets, IPRs, TADs, and inter-arched elastics to improve the precision of orthodontic movement. The disadvantages arise from the need for optimal teamwork. The patient must use the aligners for a minimum of 22 h per day. Patient collaboration guarantees the success of the ideal treatment plan.

## Figures and Tables

**Table 1:** Database search indicator.

Articles screening strategy	Keywords: orthodontics AND aligners AND distalization
	Boolean Indicators: (“A” AND “B”)
	Timespan: 10 years (2013-2023)
	Electronic Database: Pubmed, Web of Science, Scopus

**Table 2:** PICOS criteria.

Criteria	Application in the Present Study
**Population**	Both children and adults
**Intervention**	Orthodontic treatment with CAs
**Comparisons**	Comparing movements obtained with aligners
**Outcomes**	Efficacy using CAs to obtain Orthodontic movements
**Study design**	Clinical Trials

**Table 3:** A descriptive summary of each item selected is presented.

Authors and Years	Study Design	Number of Patient	Average Age (Years)	Aligners Type	Outcomes
Dai et al. (2021) [63]	Randomized clinical trial	17	25 ± 5	Invisalign aligners	Coronal movements of the maxillary and mandibular first molars, canines and central incisors were not fully achieved as expected.
R. Vaid et al. (2022) [64]	Observational study	-	-	Invisalign aligners	CAT has been shown to be biomechanically inadequate for achieving complex orthodontic movements based on aligner use alone, and it is the orthodontist’s knowledge of biomechanics that can make any aligner system succeed or fail.
Taffarel et al. (2022) [65]	Retrospective study	32	35 ± 9	Invisalign aligners	The null hypothesis that distalization of posterior teeth occurs in adult patients using Invisalign aligners was rejected. Treatment of Class II malocclusion with Invisalign aligners did not occur as expected in the virtual planning prepared by ClinCheck according to the occlusal outcome evaluation standards established by the ABO upon completion of use of a set of sequentially distalized aligners.
Sabouni et al. (2023) [66]	Case report	1	25	Invisalign aligners	The combined use of aligners with appropriate position and attachment geometry is an effective means of solving more complex orthodontic problems such as Class II malocclusions in a time frame comparable to, if not shorter than, conventional fixed orthodontics but with excellent aesthetics, oral hygiene and quality of life.
Jia et al. (2023) [67]	Observational study	-	-	CAT	Transparent aligners can effectively control the rotation and tipping of anchor units caused by 3D anchor attachment.
Al-Nadawi et al. (2021) [68]	Prospective study	80	35	Invisalign aligners	Achieving clinically similar accuracy between the 7-day and 14-day protocols in half the treatment time suggests that a 7-day protocol is an acceptable treatment protocol.
X Yan et al. (2023) [2]	Retrospective study	51	25	Invisalign aligners	For Class II division 2 patients, expected incisor proclination (69.8%) and intrusion (53.3%) are partially achieved with CAT. Excessive labial movement (0.7 mm) of the incisors may occur. Incisor movement is influenced by the amount of expected movement, premolar extraction, canine proclination, molar distalization, mini-implants, and age.
Linwei Li et al. (2023) [69]	Retrospective study	43	adults	Invisalign aligners	The efficacy of molar distalization with CAs was significantly affected by anterior teeth retraction, and the arch width significantly increased at premolar and molar levels.
Ravera et al. (2016) [70]	Retrospective study	20 (9 males and 11 females)	29.73	Invisalign aligners	Aligner therapy in association with composite attachments and Class II elastics can distalize maxillary first molar by 2.25 mm without significant tipping and vertical movements of the crown.
Auladell A. et al. (2022) [1]	Case reports	First Case (male) and second case (female)	40 and 28	Mini implants in the first case, CA in the second case	The mini-implant and the CA can be used when a correction of 2 mm or more in the sagittal plane treatment is required.
D’Antò V. et al. (2023) [41]	Prospective study	16 (4 males, 12 females)	25.7 ± 8.8	Ordoline Aligners (UABOrdoline, Vilnius, Lithuania)	The maxillary molar distalization measured at the buccal cusp tips with CAs is effective, although the clinician’s prescription, which is the ideal end-treatment goal, is no likely to be fulfilled. Therefore, refinements are necessary.
Palone et al. (2023) [71]	Retrospective study	150 (80 females, 70 males)	33.7 ± 12.7	CAT	When designing difficult movements like tilt and rotation, around 20% overcorrection should be included in the original planning phase, whereas angulation, intrusion, and extrusion needed little to no correction.
Loberto et al. (2023) [72]	Retrospective study	49 (27 females, 22 males)	14.9 ± 6	CAT	The study found significant distalization of maxillary first permanent molars, slight anchorage loss in premolars, and mesial displacement in upper canines. Transparent aligners successfully caused molar shift, but upper canine anchorage loss occurred.
Palone et al. (2022) [73]	Case report	1 female	22	Hybrid-CAT	A bone appliance was used to achieve rapid skeletal maxillary expansion and bilateral molar distalization in a patient with Class II malocclusion, maxillary skeletal transverse deficiency, and ectopic maxillary left lateral incisor.
De Felice et al. (2020) [74]	Clinical study	40	-	CAT	The study found that the actual interproximal enamel reduction (IPR) space did not match the intended amount, and less IPR was performed than anticipated, which may not be clinically significant.
Feng et al. (2022) [75]	Clinical study	21	adults	CAT	It is possible to avoid unintentional crown tilting into the extraction space during space closure by designing the distal crown tipping of the posterior teeth and the mesial crown tipping of the canines. The preliminary formula that has been provided could serve as a reference for anti-tip designs when using CAs.
Saif et al. (2021) [76]	Clinical study	38	25.4	Invisalign aligners	Invisalign is effective for adult patients requiring 2.6 mm distalization of maxillary molars, but clinicians should be aware of adverse effects, especially if the patient initially had a large overjet.
Laganà et al. (2021) [77]	Clinical study	30 (14 males, 16 females)	24.53 ± 13.41	CAT	During treatment with CAs, there is a discrepancy in the amount of interproximal enamel reduction (IPR) reported by the ClinCheck program and the amount of IPR carried out by the orthodontist.
Greco et al. (2022) [78]	Case report	1 female	25	G-Block: Posterior anchorage device TADs-supported aligners	After distalization of the maxillary molars with aligners, the use of TADs for posterior anchorage may be an efficient way to manage posterior anchorage, requiring less patient cooperation when using elastics and making movements of the posterior teeth simpler by combining the force expressed by the aligners with the force expressed by the auxiliary system.

## Abbreviations

BPA	bisphenol A
CA	clear aligners
CAD/CAM	computer-aided design and computer-aided manufacturing
CAT	clear aligner therapy
CBCT	cone beam computed tomography
EOF	extra-oral force
IPR	interproximal reduction
TAD	temporary anchorage device
DPA	difference between predicted and actual
